# Assessing higher education students’ critical thinking with the PENCRISAL test - Portuguese short version: a psychometric study

**DOI:** 10.3389/fpsyg.2023.1196794

**Published:** 2023-07-19

**Authors:** Silvia F. Rivas, Amanda Franco, Rui Marques Vieira, Leandro S. Almeida, Carlos Saiz

**Affiliations:** ^1^Psychology Faculty, University of Salamanca, Salamanca, Spain; ^2^Escola Superior de Educação de Viseu (ESEV), Viseu, Portugal; ^3^University of Aveiro, Aveiro, Portugal; ^4^University of Minho, Braga, Portugal

**Keywords:** critical thinking, reasoning, decision making, problem solving, higher education, assessment, transversal competences

## Abstract

The development of critical thinking in higher education is fundamental, preparing students to think well, find explanations, make decisions and solve problems. Given the importance of its promotion, its assessment is crucial, since the two are inseparable. Moreover, the number of instruments that are validated to assess critical thinking in the Portuguese language and culture are scarce. We present the validation psychometric study of the PENCRISAL test (short version) to the Portuguese language, a critical thinking assessment test for higher education students, designed and validated in Spain (full and short version), which presents adequate reliability and validity psychometric characteristics to assess key-dimensions of critical thinking. A sample of 225 Portuguese higher education students from three universities (two public and one private) performed a reduced version of the PANCRISAL test. The results obtained allowed replicating the Spanish reduced version in Portugal (only changing one of the six items), and the confirmatory factorial analysis permits to identify two factors intercorrelated, legitimizing the combination of the six items in a global score. This short version can be used as a screening test, and its potential is pointed out to assess students critical thinking to support teaching and research in higher education.

## 1. Introduction

Critical Thinking (CT) tends to be considered an elusive construct, given the diversity of meanings that are recognized for it in different areas (Davies, 2015). Even so, CT can be defined as a higher form of thinking that includes skills, dispositions, thinking criteria and a knowledge base, and that is useful in a diversity of life spheres, for ‘thinking well’, finding explanations, making decisions and solving problems (Franco et al., 2017c). Two core facets of CT – cognition and disposition – are found in this definition, which combine and materialize in its eminently applied character and its relevance today (Saiz, 2020).

Although CT is pointed out as transversally relevant in the various spheres of individuals’ lives, we will focus on the academic life sphere. Regardless of the year and subject area, CT is considered to be particularly important in the context of Higher Education. This is because it is precisely at this stage of psychosocial development and culmination of academic training (without considering the compelling need for lifelong learning that is now imperative) and preparation for the world – of work, of course, but also of life in society – that student-individuals are expected to prove capable of thinking critically about the diversity of information, issues, and decisions required (Franco et al., 2015; Halpern and Dunn, 2021, 2023).

The relevance of CT is widely recognised by Higher Education Institutions, both nationally and internationally, which identify the fostering of critical thinkers as one of their raison d’être (Davies, 2015; Hauke, 2019). Nevertheless, in such a purpose of CT development, it is important to consider two dimensions associated to it: its promotion and assessment. In fact, if the aim is to trigger the development of university students’ CT, it is unavoidable to dedicate time and space to its promotion – either directly, with the students themselves, and usually by infusion, in the context of curricular units; or indirectly, with their teachers, in the framework of continuing education (Franco et al., 2018b). Simultaneously, it is equally inescapable to dedicate space and time to its assessment – namely of the impact caused by the intervention for the promotion of CT (Halpern, 2016). However, the importance of CT assessment is more comprehensive. The design and construction of instruments that allow measuring the expression of students’ CT skills and dispositions, as well as their relationship with other areas of these individuals’ daily lives that go beyond the academic one, open up endless possibilities in the field of cognitive assessment and personal and social well-being (Franco and Almeida, 2015; Franco et al., 2017a,b, 2018a; Franco and Saiz, 2020). Thus, in the context of Higher Education, the assessment of the students’ CT serves the possibility of making pedagogical decisions that are better adjusted to the characteristics of the students and better guide them in their learning.

Given its relevance, in this article we will focus on the dimension of the students’ CT assessment in the specific context of Higher Education. There is a diversity of instruments to enable the assessment of CT (*cf.* Phan, 2010; Franco and Almeida, 2017). However, most of these instruments were created in the USA and were neither translated, adapted and validated for the Portuguese population – or for Portuguese-speakers –, nor thought of and designed for this culture and language. Given the absence of a reliable and validated test to assess the CT of Portuguese-speaking individuals (we refer not only to citizens of Portugal, but also to Brazilians and those from African Countries speaking Portuguese), from which data may emerge that may be compared to data from the assessment of CT in other countries, we carried out a study of the translation, adaptation and validation of the CT assessment test named PENCRISAL, starting with a Portuguese sample.

The PENCRISAL test was constructed by Rivas and Saiz (2012), teacher-researchers at the University of Salamanca in Spain. This complete version of the test was validated in a sample of more than 700 participants. Using various subsamples, several item analyzes were performed that were grouped into the dimensions corresponding to the proposed model and confirmed in the construct validity. An important fact that should be noted is the good convergent validity obtained with the Cornell test of critical thinking (*cf.* Rivas and Saiz, 2012). On the other hand, given the geographical, linguistic, and cultural proximity between Spain and Portugal, but especially given the robustness of the theoretical frameworkthat underlies it and its empirical validity, the authors proposed to conduct the translation, adaptation and validation study of the PENCRISAL test – in its short version – for Portuguese university students, so that it can be used in the development and promotion of CT among this population. More specifically, in this study we are considering a short version of the Spanish version of the test, validated before the Portuguese version and with the same number of items in both versions (Saiz et al., 2021). Original Spanish short version is formed by six items with adequate levels of reliability and validity coefficients. A sample of 340 university students from University of Salamanca was considered for its internal validation, and two factors (general reasoning and practical reasoning) have been obtained, each with 3 items. These two factors represent important dimensions on CT definition and being high correlated (*r* = 0.677) allows to consider a global score on test (Saiz et al., 2021). In this adaptation and validation for Portuguese university students, we started with the application of 20 items from the longer version of the test where the six items of Spanish reduced version are included. With this large number of items we were interested in supporting the eventual replacement of some items from Spanish version based in the analysis of scores distribution per item. A shortened or screening version allows a faster and large-scale assessment of students in classes and at the same time permits to include other variables in the assessment protocol in function of research and professional practice purposes. In any case, it’s important to assure that relevant dimensions of critical thinking are assessed and in reliability way.

## 2. Methods

### 2.1. Participants

A total of 225 higher education students took a reduced version of PENCRISAL test, aged between 17 and 47 years old (*M* = 22.88, SD = 5.47). Most of them were female (77.8%) and 51.6% were studying for a Bachelor’s degree, 43.6% for a Master’s degree, or 4.9% for a Ph.D. Students from three Portuguese universities (two public and one private) were considered: University of Aveiro (64.4%), University of Minho (25.3%) and Portuguese Catholic University (10.2%). This convenience sample answered the test between 2018 and 2020.

### 2.2. Procedures

After the identification of the PENCRISAL test, originally validated in Spain (Rivas and Saiz, 2012) and subsequently in Peru (Rivas et al., 2014), contact was made to the authors, within the framework of previous collaboration in a research project, in order to proceed with the request for its translation, adaptation, and validation for higher education students in Portugal and Portuguese speakers.

Once the authorisation was obtained, the linguistic and cultural translation of the test into the Portuguese language and culture was carried out, following recommendations in the literature in this area (Regmi et al., 2010; Borsa et al., 2012; Polit and Beck, 2014; International Test Commission, 2017) and that can be found in publications on this type of work of translation, adaptation, and validation of psychological tests (e.g., Pechorro et al., 2019; Barros and Ribeiro, 2022; Pino et al., 2022). The translation was carried out by a Portuguese researcher and a Portuguese teacher-researcher proficient in the Spanish language, and the revision of the translation was carried out by the authors of the original version of the PENCRISAL test and, additionally, by a Spanish researcher proficient in the Portuguese language. Along with the linguistic translation, the cultural translation of the instrument implied the slight adaptation of certain items, which were too strongly bound to Spanish culture, so as to be familiar to Portuguese culture and language. An example of this is item 7, which originally referred to a Brazilian woman who had moved to Spain to “give her children a better future” and which, in the Portuguese version, now refers to a Ukrainian woman who had moved to Portugal, for the same reasons as in the original item.

After the linguistic translation, and despite the cultural adaptation of the items, it was found that a group of items might not be familiar in the Portuguese culture. Consequently, by inter-judge agreement (namely the authors of the original instrument and the researcher and the teacher-researcher who intended to validate the test for Portuguese-speaking higher education students), it was decided to retain a set of 20 items from the original version of the PENCRISAL test, as they were the most representative of each factor/dimension and, simultaneously, the most appropriate to the Portuguese culture. This reduced version includes the six items of short Spanish version to be validated in Portugal. In function of scores distribution per item this large number allows us to replace any items with low variance. The preliminary Portuguese short version of the PENCRISAL test was presented to students from two Portuguese public universities and one private university, through contact made to their professors. Each student was invited to participate in the study and complete the test on an online platform, after giving informed consent.

### 2.3. Instrument

In its original extended version, the PENCRISAL test (Rivas and Saiz, 2012) has 35 open-ended items that describe problem-situations, and the respondent is asked to prepare a response explaining what she/he would decide in a given situation or how she/he would solve that situation. This test assesses a set of five dimensions of CT: deductive reasoning/deduction, inductive reasoning/induction, practical reasoning/argumentation, decision-making, and problem-solving. Each of the five dimensions is assessed from a total of seven items each. In this full version of the test, an exploratory factor analysis was performed with one of the subsamples. Based on the data obtained, a confirmatory factor analysis was performed with another subsample (*cf.* Rivas and Saiz, 2012). The validation of the short test presented in this work is based on these data. More detailed information on the identified CT dimensions can be found in Rivas and Saiz (2012) and Saiz and Rivas (2008).

The administration of the PENCRISAL test is carried out through an online platform with internet connection, which each respondent accesses (in a single session or, as recommended, in several sessions) by entering a unique access password, created on the basis of her/his student ID number. Although no time limit is given for the PENCRISAL test, the total time taken varies between 60 and 90 min.

As far as their scores are concerned, 0, 1 or 2 points are awarded for each open answer provided by the respondent, depending on whether the answer is less or more precise/complete, respectively. Specifically, 0 points are awarded for an answer that is incorrectly resolved, 1 point for an answer that is correctly resolved but they lack justification or explanation of why they respond that way, and 2 points for an answer that is correctly resolved and it presents a justification or explanation of why they respond that way. In the total score, the possible range of the PENCRISAL test varies between 0 and 70 points, and this value varies between 0 and 14 points in each of the five dimensions assessed by the instrument.

A shortened version of PENCRISAL exists for Spanish students. This version consists of six items, three assessing general reasoning and the other three assess practical reasoning, understood as two relevant dimensions in the definition of critical thinking (Saiz et al., 2021). In this study a version of 20 items was applied (four items per dimension), where those six items of Spanish reduced version have been intentionally included.

## 3. Results

Before testing factor structure of six items from short Spanish version, an exploratory analysis of the data was conducted to appreciate the variance or dispersion of students scores in each item. This analysis showed that in several items the students did not answer correctly, even partially, and obtain zero points. After that, it was necessary to replace item 3 (practical reasoning/argumentation) from Spanish short version since it did not show enough variation among the Portuguese students. Based on this descriptive analysis, the item 15 (general problem solving) was chosen to replace item 3 (practical reasoning/argumentation). On Table 1 the six items of Spanish and Portuguese version to be tested are present. As we can see, the three items of *general reasoning factor* are the same in both versions, and in *problem-solving and practical reasoning* factor the item 03 (practical reasoning) in Spanish version was substituted by item 15 (general problem solving) in Portuguese version.

**Table 1:** Items in Spanish short version and in Portuguese version to be tested.

	Factor 1: *General reasoning* (3 items)	Factor 2: *Problem-solving and practical reasoning* (3 items)
Portuguese version	Item 04 - Propositional Reasoning Item 14 - Analogical Reasoning Item 18 - Practical Reasoning/Argumentation	**Item 15** - General Problem Solving Item 19 – Fallacy Item 20 – Fallacy
Spanish version	Item 04 – Propositional Reasoning Item 14 – Analogical Reasoning Item 18 – Practical Reasoning/Argumentation	**Item 03** – Practical Reasoning/Argumentation Item 19 – Fallacy Item 20 – Fallacy

In bold, the only divergence in data between the two versions.

On the one hand, factor 1, with three items, incorporating items 4, 14, and 18, related to the general reasoning dimension; on the other hand, factor 2, also with three items, including items 15, 19, and 20, related to the problem-solving and practical reasoning/argumentation dimension (*cf.* Table 1); and item comparison table in all versions in: https://www.pensamiento-critico.com/archivos/tableitems6EN.pdf it should be noted that the difference between the Spanish and the Portuguese short versions lies in one item. Item 15 (captured by the Portuguese version) refers to problem-solving, whereas item 3 (captured by the Spanish version) refers to propositional reasoning. The difference between both CT dimensions may seem clear, although it is not necessarily so. Item 15, referring to problem-solving, involves general problem-solving strategies, not specific strategies. It is important to note here that all items in the PENCRISAL test concern everyday problems, so that in each one it is necessary to solve the situation in a differentiated way. In situations related to reasoning, as in item 3 of the Spanish version, a conclusion must be reached, which inevitably represents a decision or a solution offered to the problem at hand. There is an equivalence between conclusion, decision, and solution, which sometimes eludes the individual’s understanding, who does not always see or understand it (Saiz, 2020). For this reason, although items 3 and 15 belong to distinct CT dimensions, item 15 – of general problem-solving – resembles item 3 by the fact that both translate general forms of problem-solving. However, the fact that no coincidence was detected between the two short versions of the PENCRISAL test in these items may result from some difference between the two populations (Portuguese and Spanish). In any case, the similarity of the format of the items – presented as everyday problems – may have led both items to be perceived as being of the same type, given their content.

**Figure 1:**
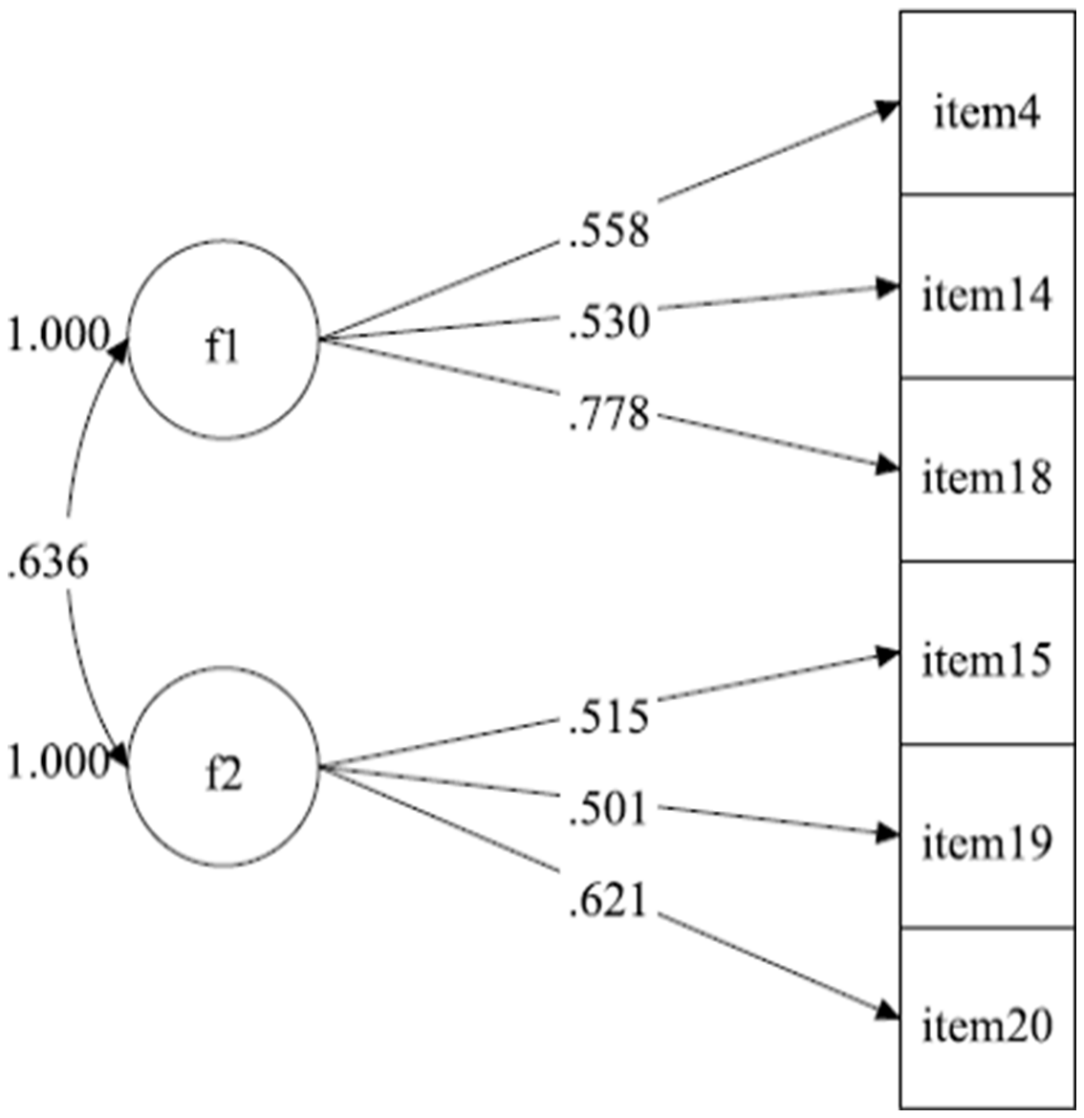
Factor structure of the six items in Portuguese short version.

After this change in one item, and maintaining basically the same test version, we performed confirmatory factor analysis (CFA), testing the model of two correlated factors already validated in the Spanish population (*cf.* Saiz et al., 2021). For CFA, the programme M-Plus (v. 8.6, Muthén and Muthén, 2019) was used due to the essentially ordinal nature of the items (even ranging between 0 and 2 points), the estimators used in the CFA were the mean and the weighted least- square means and variances (WLSMV). The indexes taken to determine the quality of the model fit were those recommended for this type of analysis in the literature: X-square (χ2)/df < 3.0; Comparative Fit Index (CFI) and Tucker-Lewis Index (TLI) > 0.90; Root Mean-square Error of Approximation (RMSEA) < 0.08; and Standardized Root Mean Square Residual (SRMR) < 0.05 (MacCallum et al., 1996; Hu and Bentler, 1999; Kaplan, 2000). More specifically, the CFA performed revealed adequate fit indices: *χ*^2^/df = 7.01; CFI = 0.91; TLI = 0.90; RMSEA = 0.07; and SRMR = 0.039. Figure 1 shows the factor weights of the six items divided by the two factors isolated in the analysis.

As can be seen (*cf.* Figure 1), the six items captured in the Portuguese short version of the PENCRISAL test are satisfactorily saturated in each factor (showing saturation indices between 0.501 and 0.778). As regards the correlation between the two emerging factors – General Reasoning and Problem-Solving and Practical Reasoning/Argumentation –, it shows a somewhat high value (*r* = 0.636), which means that both converge towards the more general construct assessment that we call CT. In this sense, it becomes legitimate to consider students’ scores in each of the two factors, as well as their score by adding up the scores in both factors, as in Spanish version (correlation between two factors was 0.677). In Table 2 the loadings of six items in each factor are presented, showing values higher 0.40 in Spanish version and higher 0.50 in Portuguese version.

**Table 2:** Loadings of six items in each factor for both Spanish and Portuguese versions.

	Spanish version		Portuguese version	
Items	Factor 1	Factor 2	Factor 1	Factor 2
Item 04 - Propositional Reasoning	0.486		0.558	
Item 14 - Analogical Reasoning	0.473		0.530	
Item 18 - Practical Reasoning/Argumentation	0.551		0.778	
**Item 03** – Practical Reasoning/Argumentation		0.473		–
**Item 15** - General Problem Solving		–		0.515
Item 19 – Fallacy		0.437		0.501
Item 20 – Fallacy		0.487		0.621

Considering the students’ performance in the two factors separately and as a whole, the descriptive indices of the distribution of results were analysed, such as minimum and maximum values, mean and standard deviation, and also the skewness and kurtosis coefficients of the distribution (*cf.* Table 3).

**Table 3:** Distribution scores in the two factors and general factor.

Dimensions	Min.-Max.	Mean	SD	Skewness	Kurtosis
Factor 1	0.0–4.0	1.36	0.83	0.683	0.591
Factor 2	0.0–6.0	1.91	1.61	0.412	−0.806
General Factor	0.0–8.0	3.28	2.04	0.251	−0.769

The values obtained reveal that the students presented a lot of difficulties in the tests, as the score in each item varied between 0 and 2 points. For example, taking the set of six items, the maximum score would be 12.0 points, whereas the value in this sample was 8.0, and the average of the scores in the set of six items was only 3.28. High level of difficulty was also observed in both separate factors. It should be added that, even so, the values for skewness and kurtosis are lower than unity, which suggests a Gaussian distribution of the results, both in the two factors and in the general factor grouping the other two.

## 4. Discussion

Taking into account the objective of obtaining, from the PENCRISAL test, a reduced version capable of being applied in a shorter period of time and in larger samples of students, we can point out that this objective was met. Through the CFA, it was possible to establish three items for two factors, which, as they are highly correlated, allow for the formation of a global score taking into account the six selected items.

Reflecting on the theoretical basis of the emergent factor structure, although this solution contains only six items (*cf.* Table 1), these retained items capture core CT skills related to inductive reasoning/induction, deductive reasoning/deduction, and practical reasoning (three of the five dimensions of the original short version of the PENCRISAL test, explained above), which, when taken as a whole, illustrate the most representative CT skills. Let us analyse the nature of the items incorporated in each type of reasoning or factor in the short Portuguese version of the PENCRISAL test.

With regard to General Reasoning (factor 1), the items that integrate it concern (i) propositional reasoning, (ii) analogical reasoning, and (iii) practical reasoning/argumentation (item 4, item 14, and item 18, respectively). In other words, the factor captures forms of inference present in everyday cognitive functioning, from the simplest (propositional reasoning), to the quite frequent ones used when the individual cannot be more precise in her/his reasoning (analogical reasoning), to the most complex ones (practical reasoning/argumentation; Saiz, 2020; Saiz et al., 2021). (i) With propositional reasoning, referring to deduction and explanation, the individual’s objective is to test the hypotheses formulated, in order to find a plausible explanation for phenomena. Deduction is the only way to establish certain, non-provable conclusions, or, as logicians like to say, the only way to reach necessary truths. (ii) With analogical/causal reasoning, essentially referring to induction (by the nature of its conclusions, although referring to deduction by its structure), the individual’s objective is to determine how robust a conclusion is, and to do so uses an analogy that facilitates the thinking process. Whenever we say ‘it’s as if…’ or equivalent expressions, we are reasoning analogically. (iii) With practical reasoning/argumentation, the individual’s objective is to organize the information available to her/him regarding a given topic, looking for reasons/assumptions that lead to the conclusion and support it, in order to make a diagnosis (*cf.* Saiz, 2020).

With regard to Problem-solving and Practical Reasoning (factor 2), the items within it refer to (i) general reasoning (item 15), and (ii) fallacies (items 19 and 20). In general terms, general reasoning aims at solving problems or achieving an intended objective, whereas fallacies refer to forms of invalid argumentation used in everyday life. (i) With general reasoning, the individual’s objective is, fundamentally, to solve problematic situations of an ecological nature – such as those illustrated by the items of the PENCRISAL test – from eight steps that must necessarily be followed in that order if we are to proceed properly (Saiz, 2020): consider the context; observe; examine the type of evidence; delimit the motive; collect data personally; build the likely causal scenario; determine the complete meaning of the phenomenon-problem; and make a prognosis. (ii) With regard to fallacies, in the context of argumentation, the individual’s objective is to detect the arguments that are not truly so, i.e., that have no validity and that, therefore, should be identified and deconstructed, avoiding being persuaded by them (Saiz, 2020).

## 5. Conclusion

Given its academic relevance, but mainly due to its transversal relevance in the various spheres of life in which each individual moves, all efforts to assess the CT of Higher Education students – in order to promote it – continue to be welcome. This article, in which the psychometric validation study of the short Portuguese version of the PENCRISAL test is presented, intends to ensure a contribution to that objective. In addition to its value as an instrument to capture central dimensions of CT in the Portuguese language and culture, this instrument will prove to be essential to continue characterising the expression of CT across countries. In fact, the PENCRISAL test (in its extended version, with 35 items) has already been validated in Spain (*cf.* Rivas and Saiz, 2012) and also in Peru (*cf.* Rivas et al., 2014), thus this short Portuguese version offers not only an opportunity for transnational application, but also an opportunity to assess the CT in a faster way, joining the validation study of the Spanish short version (*cf.* Saiz et al., 2021).

We cannot know the absolute potential of each one, but we can measure the degree of expression of each one. This is what we can deal with (Saiz, 2017). And such to promote opportunities to mobilize and develop the CT capacities and dispositions of all students. The Portuguese short version of the PENCRISAL test is a useful screening tool for teachers and researchers interested in knowing the cognitive characteristics of their students and how their critical thinking skills can be developed throughout their academic training or be used in the promotion of pedagogical practices that meet the students needs.

However, we must recognize that some methodological aspects can be improved and that we describe below. The short version of the test (6 items in this proposal) is suitable for a rapid assessment of critical thinking skills in large samples of students in different educational research projects. However, we must delve into future studies regarding its precision and psychometric validity. In this sense, it is convenient to use more current and powerful psychometric analysis techniques, for example, item response theory (IRT). In any case, our short version allows the integration of other psychological and academic variables of the students into the evaluation protocol in order to investigate the effects of these variables on academic performance using structural equations.

## Data availability statement

The raw data supporting the conclusions of this article will be made available by the authors, without undue reservation.

## Ethics statement

Ethical review and approval was not required for the study on human participants in accordance with the local legislation and institutional requirements. The patients/participants provided their written informed consent to participate in this study.

## Author contributions

SFR and CS: conceptualization, research, and software. SFR and LA: data curation, formal analysis, and methodology. SFR, AF, RV, and LA: resources and writing – review and editing. SFR, CS, and LA: supervision, validation, and writing – original draft. All authors contributed to the article and approved the submitted version.

## Funding

This article, framed within the RV postdoctoral research project (SFRH/BPD/122162/2016), was funded by National Funds through FCT - Fundação para a Ciência e a Tecnologia, I. P. (Foundation for Science and Technology), under project UIDB/00194/2020.

## Conflict of interest

The authors declare that the research was conducted in the absence of any commercial or financial relationships that could be construed as a potential conflict of interest.

## Publisher’s note

All claims expressed in this article are solely those of the authors and do not necessarily represent those of their affiliated organizations, or those of the publisher, the editors and the reviewers. Any product that may be evaluated in this article, or claim that may be made by its manufacturer, is not guaranteed or endorsed by the publisher.
